# Characterization of the mitochondrial *Huso huso* genome and new aspects of its organization in the presence of tandem repeats in 12S rRNA

**DOI:** 10.1186/s12862-023-02166-2

**Published:** 2023-09-26

**Authors:** Khadijeh Dadkhah, Ghodrat Rahimi Mianji, Ali Barzegar, Ayoub Farhadi

**Affiliations:** 1 Laboratory for Molecular Genetics and Animal Biotechnology, Faculty of Animal Sciences and Fisheries, Sari Agricultural Sciences and Natural Resources University, Sari, Iran; 2 Department of Basic Sciences, Sari Agricultural Sciences and Natural Resources University, Sari, Iran

**Keywords:** *Huso huso*, Mitogenome, Sturgeon, Caspian Sea, Tandem repeats

## Abstract

**Background:**

The sturgeon group has been economically significant worldwide due to caviar production. Sturgeons consist of 27 species in the world. Mitogenome data could be used to infer genetic diversity and investigate the evolutionary history of sturgeons. A limited number of complete mitogenomes in this family were sequenced. Here, we annotated the mitochondrial *Huso huso* genome, which revealed new aspects of this species.

**Results:**

In this species, the mitochondrial genome consisted of 13 genes encoding proteins, 22tRNA and 2rRNA, and two non-coding regions that followed other vertebrates. In addition, H. huso had a pseudo-tRNA-Glu between ND6 and Cytb and a 52-nucleotide tandem repeat with two replications in 12S rRNA. This duplication event is probably related to the slipped strand during replication, which could remain in the strand due to mispairing during replication. Furthermore, an 82 bp repeat sequence with three replications was observed in the D-loop control region, which is usually visible in different species. Regulatory elements were also seen in the control region of the mitochondrial genome, which included termination sequences and conserved regulatory blocks. Genomic compounds showed the highest conservation in rRNA and tRNA, while protein-encoded genes and nonencoded regions had the highest divergence. The mitochondrial genome was phylogenetically assayed using 12 protein-encoding genes.

**Conclusions:**

In H. huso sequencing, we identified a distinct genome organization relative to other species that have never been reported. In recent years, along with the advancement in sequencing identified more genome rearrangements. However, it is an essential aspect of researching the evolution of the mitochondrial genome that needs to be recognized.

**Supplementary Information:**

The online version contains supplementary material available at 10.1186/s12862-023-02166-2.

## Background

Studying the molecular genetics of sturgeon could provide an excellent way to identify their origin and uncertain aspects. The *Acipenseridae* consists of four genera (*Acipenser*, *Huso*, *Pseudoscaphirhynchus*, and *Scaphirhynchus*), of which there are 25 species. Two genera, *Acipenser* and *Huso*, inhabit the Caspian Sea. Most sturgeons have been identified as endangered by the International Union for Conservation of Nature (IUCN) [1]. Studies have shown that caviar production has declined in Europe and North America, as well as in Russia and Iran [2]. Sturgeon is one of the most valuable groups of fish due to caviar production. Sturgeons are very important in genetic matters due to their 200 million-year-old history; it has been known as living fossil since the Jurassic era. Caspian sturgeon are listed in the CITES Convention. The sturgeon population is endangered for problems such as irregular and illegal fishing of sturgeon, environmental changes, climatic conditions, low rate of artificial reproduction, loss of natural environments, and pollution.

Molecular studies on the genetic structure could help to identify valuable information about this endangered fish. In determining the genetic structure of Caspian sturgeon, molecular techniques such as RFLP [3], RAPD [4], AFLP [5–7], microsatellite [8–12], and DNA sequencing [13, 14] have replaced traditional methods such as meristics and morphometrics. DNA sequencing is one of the most accurate methods of identifying information about sturgeon [15]. Mugue et al. [15] distinguished sturgeon at the species level with mitochondrial D-loop sequencing. In another study, mitochondrial D-loop sequencing was used as a complementary method to other laboratory methods [16]. Mitochondrial genome sequencing is a simple, immediate, and reliable method to identify species. The Cytb and control regions have been used mainly to identify sturgeon species. However, researchers have found that the Cytb gene does not have a good resolution for identifying these species [15]. The size of mitochondrial DNA is approximately 15–20 kb, and the mitochondrial genome contains several copies, is double-stranded, and has a circular molecule. It contains 13 protein-encoding genes, two genes encoding ribosomes, 22 genes encoding tRNAs, and two control regions. Because mitochondrial genomic DNA is inherited from the mother [17], it is suitable for evolutionary and historical studies [18]. Ludwig et al. [19] illustrated VNTR duplicate blocks in some sturgeon species in the control region in region 5' immediately after proline tRNA, which was three replicates with a size of 82 bp. Mugue et al. [20] also reported a repetitive sequence of 82 pairs of bases in the ship specie. Research has shown that repetitive blocks in the control region are related to genes close to CR (12S rRNA and tRNA) [21].

Ciftici et al. [22] recognized the presence of duplicate blocks in the D-loop regions and reported an 82–83 bp tandem repeat by sequencing in *Acipenser gueldenstaedtii*, *Acipenser stellatus*, and *huso huso* species. The frequency of multiple copies of the genome in a species may be attributed to the differences in mutation rates or a mechanism that may regulate mutation rates. A large number of studies have been performed on the teleosts fish of control regions that identified VNTR [19, 20, 22]. However, in this study, we also detected the presence of tandem repeats in the other two regions, 12S rRNA and tRNA. Previous research has shown excess tRNA in several fish species [21–26], but there have been no reports about the presence of repeats in 12 SrRNAs.

The vertebrate mitogenome is highly conserved; however, with increasing genome sequence data for fish, reports of rearrangement have been observed. The present study aims have been to draw a complete map of the mitochondrial genome of *Huso huso* and a complete sequencing of the mitochondrial genome, determination of genetic structure and phylogenetic relationships, and codon usage. Our attempt initially was to study the mitochondrial genome and its molecular mechanisms; in the following, we found novel aspects of rearrangement in this species. This study reveals that the mitochondrial genome of sturgeon could have differed in organization, gene content, and order. In addition, these results will provide a better perspective of understanding fish's evolution.

## Results and discussion

### Genome organization

The complete mitogenome of *Huso huso* was deposited in the Gene Bank (Accession number: MK213068). The *Huso huso* mitochondrial genome consisted of 16836 bp. This genome contained 37 encoding genes, 13 genes encoding proteins, 22tRNA and 2rRNA, and two OL and D-loop control regions, in which an additional tandem repeat tRNA- Glu was observed in Table 1.

**Table 1:** Summary of gene features of *Huso huso*

Gene	Coding	Position		Size (bp)	Codon		Anticodon	Intergenic
								Nucleotides
	Strand	From	To		Start	Stop		
tRNA Phe	H	1	68	68			GAA	
12SrRNA	H	69	1081	1013				0
tRNA val	H	1082	1151	70			UAC	0
16SrRNA	H	1153	2854	1702				+ 1
tRNA leu	H	2855	2929	75			UAA	0
ND1	H	2930	3904	975	ATG	TAG		0
tRNA Ile	H	3914	3984	71			GAU	+ 9
tRNA Gln	H	3984	4054	71			UUG	-1
tRNA met	L	4054	4123	70			CAU	0
ND2	H	4124	5168	1045	ATG	TAG		0
tRNA trp	H	5169	5241	73			UCA	0
tRNA ala	H	5243	5312	70			UGC	+ 2
tRNA asn	H	5314	5386	73			GUU	0
OL	L	5387	5420	34				0
tRNA cys	L	5421	5487	67			GCA	0
tRNA tyr	L	5488	5558	71			GUA	0
COI	L	5560	7128	1569	GTG	TAA		+ 1
tRNA ser	H	7120	7192	73			UGA	-7
tRNA Asp	L	7198	7269	72			GUC	+ 7
COII	H	7284	7974	691	ATG	T		+ 14
tRNA lys	H	7975	8048	74			UUU	0
ATP8	H	8050	8217	168	ATG	TAA		+ 1
ATP6	H	8208	8891	684	ATG	TAA		-8
COIII	H	8891	9675	785	ATG	TAA		0
tRNA Gly	H	9676	9748	73			UCC	0
ND3	H	9749	10097	349	ATG	TAG		0
tRNA Arg	H	10098	10167	70			UCG	0
ND4 L	H	10168	10464	297	ATG	TAA		0
ND4	H	10458	11838	1381	ATG	T		-5
tRNA His	H	11839	11907	69			GUG	0
tRNA ser	H	11908	11975	68			GCU	0
tRNA leu	H	11976	12048	73			UAG	0
ND5	H	12049	13890	1842	ATG	TAA		0
ND6	L	13887	14408	522	ATG	TAG		-2
tRNA Glu	L	14409	14478	70			UUC	+ 4
tRNA Glu	L	14483	14552	70			UUC	0
cytb	H	14555	15695	1141	ATG	T		+ 2
tRNA thr	H	15696	15769	74			UGU	0
tRNA pro	L	15773	15842	70			UGG	+ 3
D-loop	H	15842	16836	994				0

**Fig. 1:**
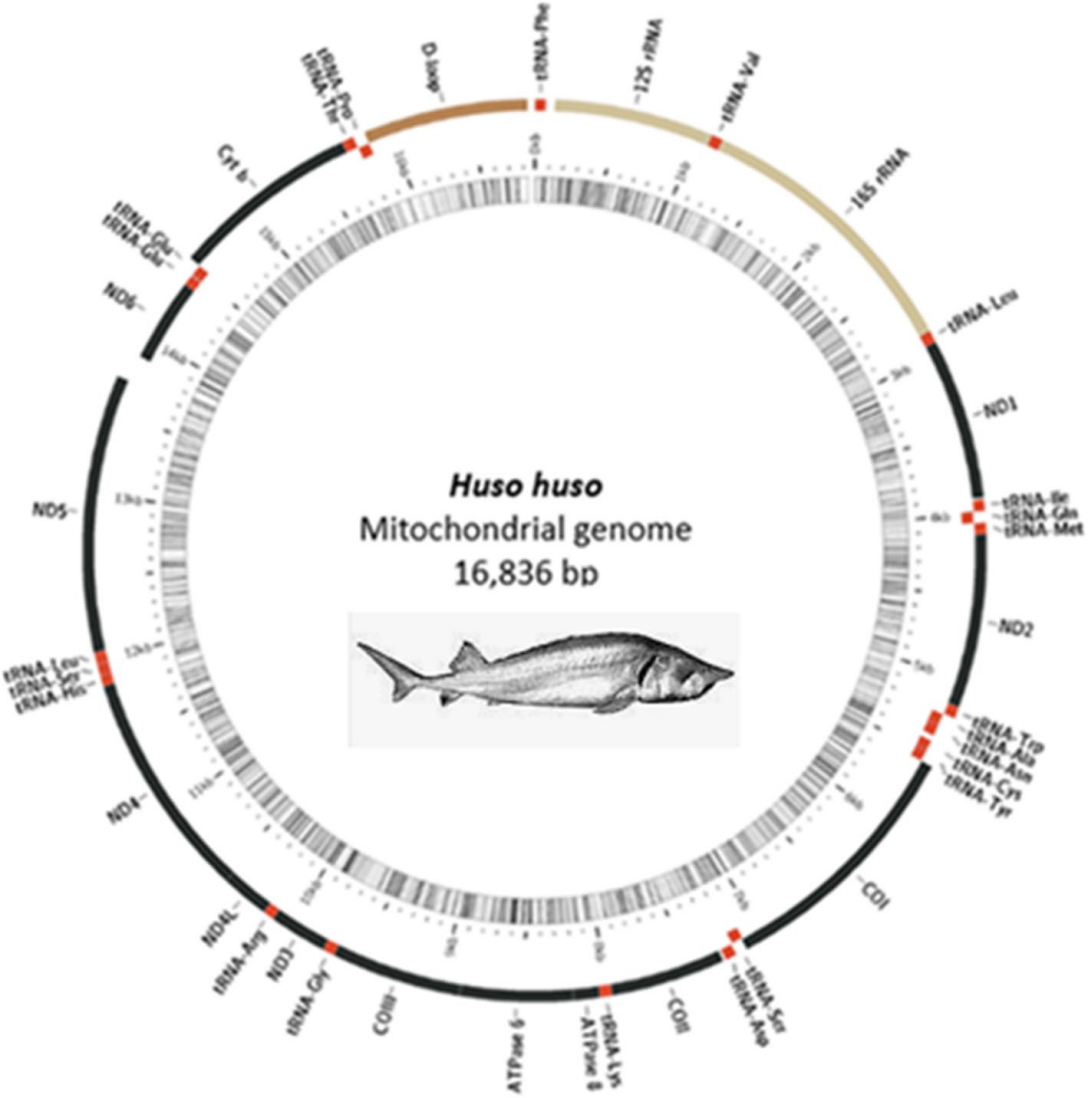
The complete mitochondrial genomes in *Huso huso*. Protein coding, ribosomal RNA, and transfer RNA genes are shown using different colors. Genes encoded on the H-strand are in the outer region. Genes coded on the L-strand are in the inner region

Like many mitochondrial genomes, most genes were located in the heavy strand except ND6 and eight tRNA (tRNAGLN, tRNAala, tRNAGLU, tRNAser, tRNA tyr and tRNAcys, tRNA Asn and tRNA Pro) ‏that were coded in the light strand. The D-loop (displacement loop) was located in the main non-coding region of the mitochondrial DNA molecule. The mitochondrial DNA could be replicated in two different ways, starting in the D-loop region. OL is the origin of light strand replication, which was identified in the WANCY region with a cluster of five tRNA similar to that of other vertebrates. There were two tRNA-Glu with repeated sequences, which could be seen in Fig. 1.

Nucleotide asymmetry of the strand is usually described by AT, and GC skews. GCskew is barely above zero (they are mostly negative). These results illustrate that the content of A is only slightly higher than that of T, whereas that of C is significantly higher than that of G. Skews are related to the difference in mutation pressures applied in light and heavy strings [27]. As a result, they are asymmetric and cause changes in mtDNA [18, 28, 29].

### Genome organization and evolutionary mechanism

A new type of mitochondrial genome organization has been found to contain an additional tRNA. This extra tRNA can be folded, creating a clover leaf structure of tRNA that also contains an anticodon.

This tRNA is very similar to the conventional tRNA sequence and is located between ND6 and Cytb. This issue is a new genome organization, and it could create new specific features in the genus *H. huso* that distinguish it from other sturgeon species. Changes in the tRNA of other fish species have also been observed [21–25]. In a study of a fish species, additional tRNA-Ile was generated with anticodon mutations TAC to AAT. It was also seen in *Serranidae* that the extra tRNA-Asp in the light strand resulted from the rearrangement of the mitochondrial genome [30, 31].

This gene content of the fish mitochondrial genome is typically the result of gene amplification and causes diversity in species.

The rearrangement of the mitochondrial genome observed in fish usually can include translocation [32, 33], which is also due to the tandem duplication of gene regions. These results showed that extra tRNA is left in the mitochondrial genome after changes. Several abnormal fish mitochondrial genome tRNAs include extra tRNA-ser at the downstream ND5 in sea bass *Morone saxatilis* [24], extra tRNA met in *Pampus species* [22], the pseudo tRNA at the same position in parrotfish *Chlorurus sordidus* [23], extra tRNA Asn, pseudo tRNA ala in the WANCY cluster polar cod *Boreogadus saida* [21], and tRNA pro amplification in CR from *Antarctic notthenioig* [25] were found in previous results. These reports have shown that new tRNAs appear to have been seen in various places. This issue results from rearrangement and mispairing, which ultimately leaves one or more additional tRNA in the mitochondrial genomes of different fish species. Rearrangements in bony fish determine the complete nucleotide sequence of the mitochondrial genome [34], such that for a benthic fish, *Gonostoma gracile*, the entire mitochondrial genome includes 19 tRNA genes that exist in typical vertebrates. However, the gene sequence of tRNA Glu is different [35].

### Protein coding genes

The cumulative length of *H. huso* mitochondrial protein-coding genes was 11406 bp, which was calculated as 67% of the total length of the mitochondrial genome. The genes encoded in mtDNA were highly compact and contained overlapping sections. Our overlap was between 13 protein-encoding genes in this species between ATP8 and ATP6, which had an eight bp overlap, between ND4 L and ND4, which was five bp, and between ND5 and ND6, it was found to be two bp.

Most genes encoding mitochondrial proteins in this species began with the ATG primer, similar to many metazoa [36]. The COI gene only had one separate start codon, GTG. Among the coding genes for the protein, the Cytb, COII, and ND4 genes were terminated in T (Table 1). This incomplete codon is completed with the addition of poly A and finally will become TAA.

The calculation of available nucleotide abundance at each codon position in all 13 protein genes is shown in the table. The T nucleotide was mostly seen in the position of the second codon. Since triple codons are encoded with T in the second position for hydrophobic or hydrophobic residues, this observed deviation indicates a high ratio of hydrophobic residues between the coding proteins. The 12 genes encoding proteins in the heavy strand shared an anti-G deviation; it was approximately 10% or less at the third codon position. The deviation, primarily in the position of the third codon, is probably due to the selection pressure on the synonymous mutation in this position (Table S2).

**Fig. 2:**
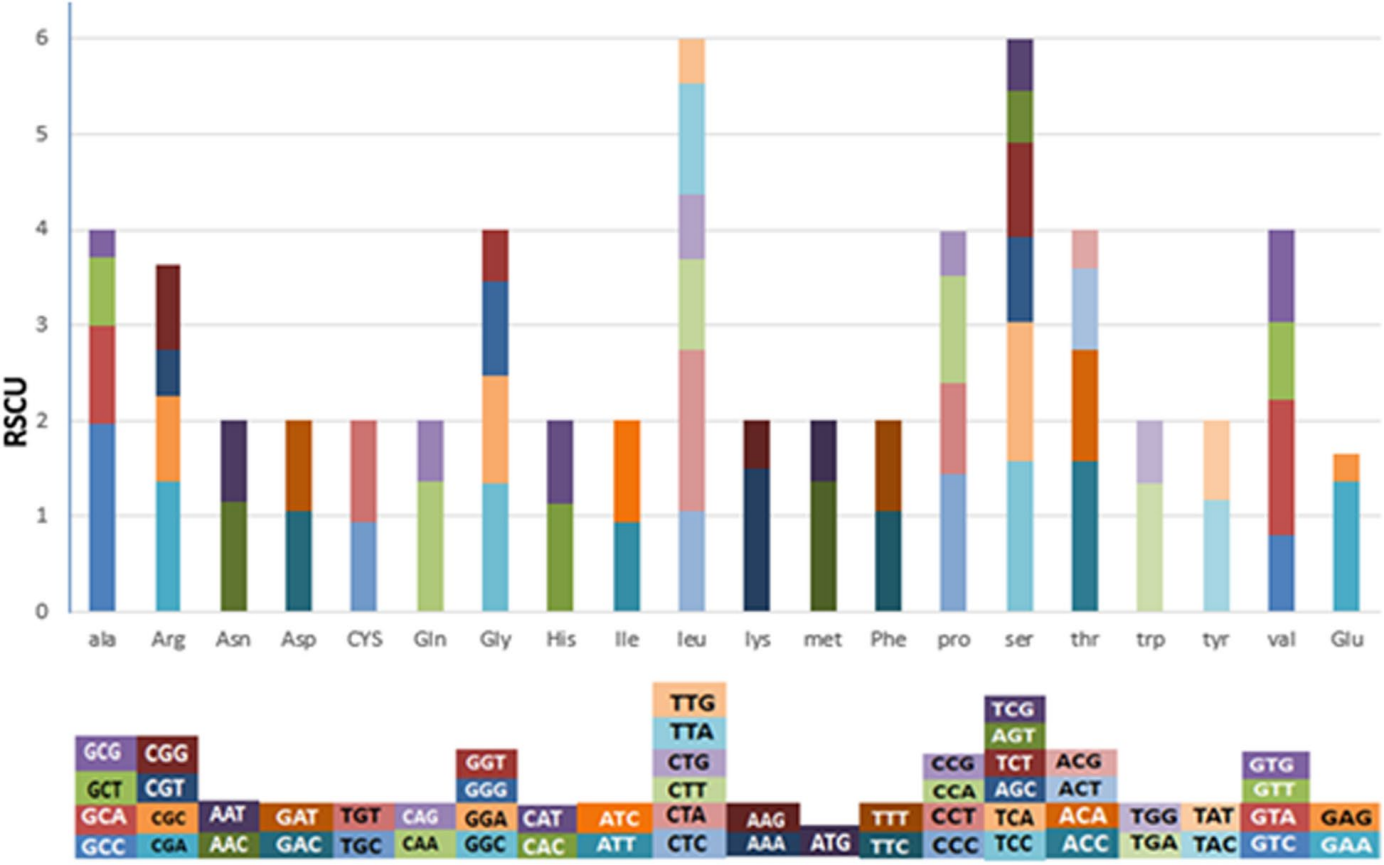
Relative synonymous codon usage (RSCU) of the mitochondrial protein-coding genes and codon usage of *Huso huso* for whole genome sequencing

Serine and leucine amino acids indicated the highest frequency in *Huso huso*, using six different codons, while some amino acids used 2 or 4 codons. After them, the amino acids alanine, threonine, glycine, proline, and valine showed the highest frequency with four codons. Of course, arginine also had four codons and was observed with a relatively high frequency. The rest of the amino acids used two codons, which displayed the same frequency (Fig. 2) (Table S3).

#### rRNA and tRNA

Evaluation of rRNAs in this species showed that the length for 12S rRNA was 1013 bp and for 16S rRNA was 1702 bp. The H strand encoded both rRNAs, and their nucleotide composition for 12S rRNA and 16S rRNA was A% 31.4, C% 27.5, T% 19.3, G% 21.7, and A% 35.5, C% 24.6, T% 19.6, G% 20.0, respectively. The deviation of the nucleotide composition is a strand-specific property in mtDNA, and this deviation observed is against G. Similar phenomena have been found in the mitochondrial genome of other fishes [37] and mammals [3] that are thought to be related to asymmetric replication of the H and L strand [38, 39], exceptionally in stem 16S rRNA and 12S rRNA, which is necessary for stability in the stem structure, and the deviation against G observed less [4, 40, 41].

tRNAThr and tRNAPro relative to cytochrome b differ from those determined in other vertebrates.

In region 5′ 12S rRNA, a 52-nucleotide VNTR sequence was observed with the following sequence, which could be the result of mispairing:

- AGGCTTGGTCCTGGCCTTACTATCAATTTTAACCCAATTTACACATGCAAGT

**Fig. 3:**
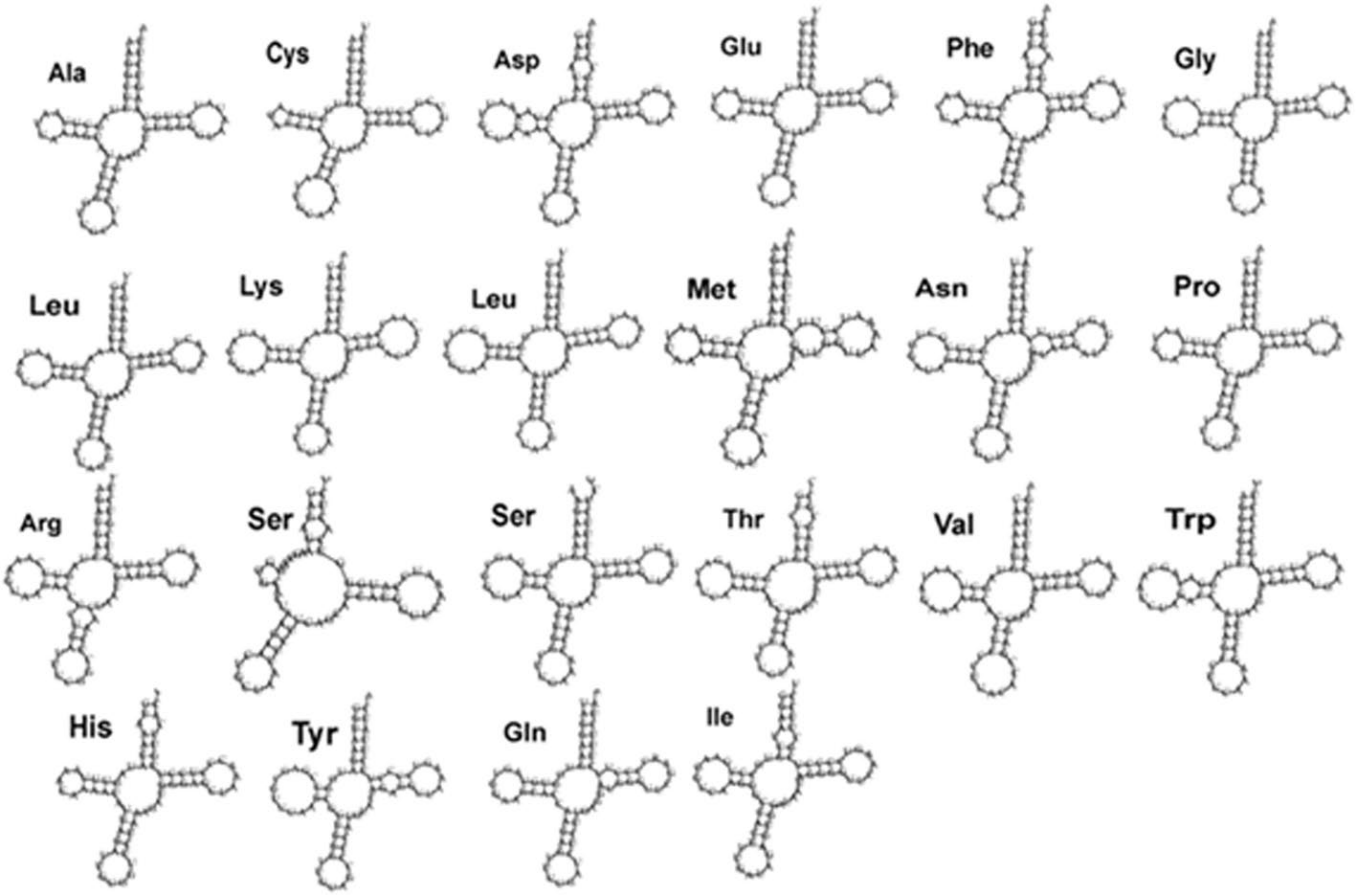
The secondary structure of tRNA genes in *H.huso*

This sequence could produce stems, loops, and a stable structure (Fig. S1). Of the tRNAs, 14 were encoded by the heavy strand, these tRNAs are 67 to 75 bp. All tRNAs except ser (AGY) could be converted to clover leaves, the structure of which could be determined by tRNA scanning. tRNAser was reduced in the dehydrooridine arm, transforming its second structure into a short clover leaf, similar to most metazoa [42] (Fig. 3).

#### Noncoding regions

Noncoding regions in mtDNA include OL and CR and several regions between genes. The size of the CR has been 994 bp, which is located downstream of tRNAper. This region is rich in AT and is considered the largest noncoding region.

CR contains several TASs, and the TAS sequence extends into several CR regions. This sequence is associated with CSB-conserved sequence blocks. The conserved CSB-1–3 sequence blocks are downstream of this region. The TAS region is rich in repeats and is the most variable part of the CR. It contains duplicate elements with a length of 82 bp. It contains a conserved TACAT motif, and the TAS motif is an inverse complement to ATGTA. The TAS motif can be paired with the CTAS motif, resulting in the formation of stable hairpin loops, which may also serve as a specific sequence signal to terminate mtDNA replication [43]. Repeat sequences were identified in this region in fish of different species [44–46].

**Fig. 4:**
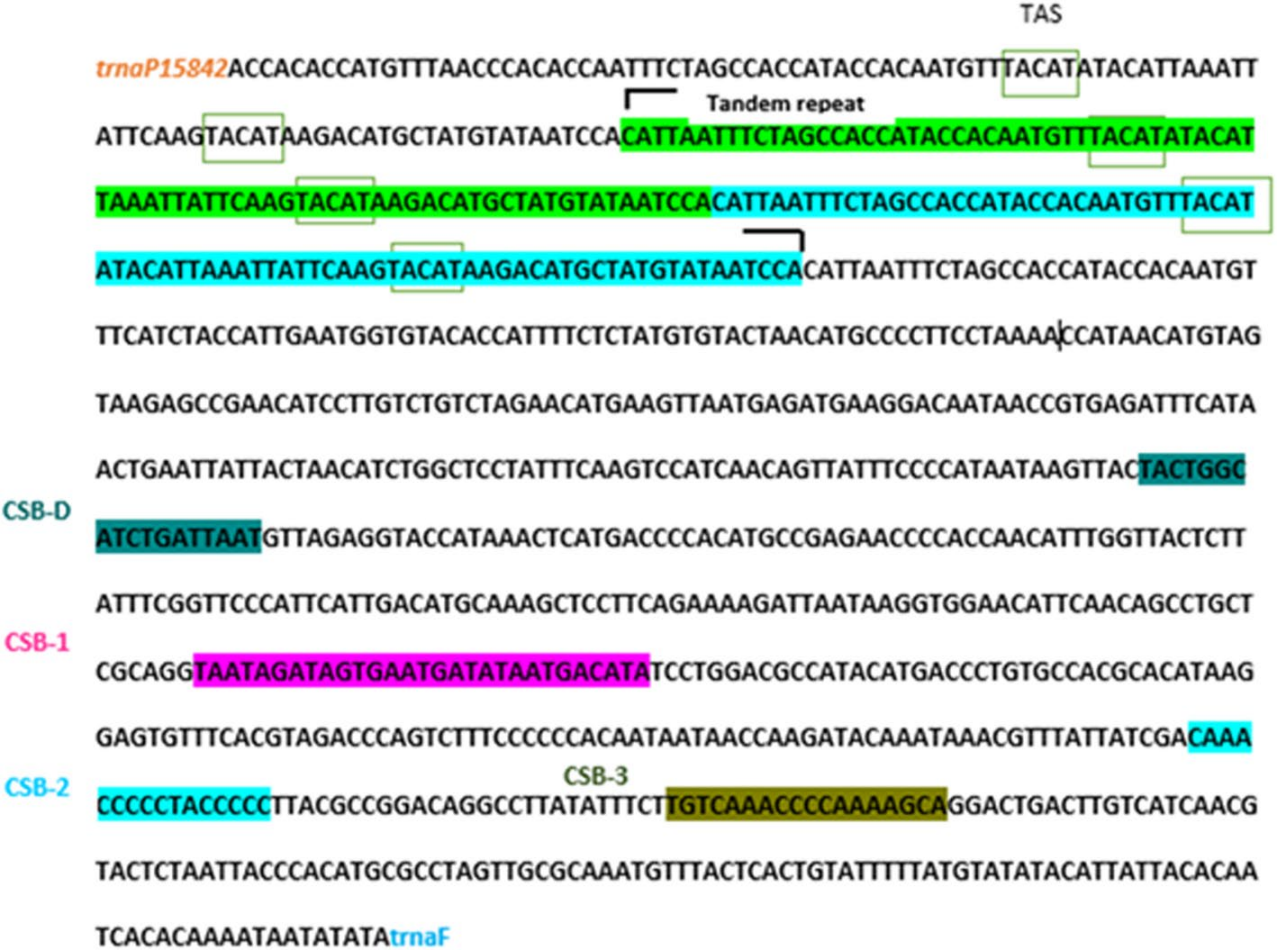
The D-loop region, along with the CSB-conserved blocks

A comparison of mtDNA sequences in sturgeon and conserved sequences of termination replication (TAS) sturgeons with changes in the number of consecutive duplicate sequences showed that they could form stable structures during mtDNA replication. In the mtDNA of sturgeons, the control region responsible for the termination of H-strand replication contains one to seven variable number tandem repeats (VNTRs) with a unit size of 78 to 83 bp [47]. The D-loop of sturgeon mtDNA differs from the human D-loop and contains more than one TAS; therefore, the termination of mtDNA replication cannot be explained by helicase activity alone. Additionally, it is not still determined why sturgeons exhibit a wide range of haplotypes differing in VNTR length and, correspondingly, in the number of TAS elements [47]. Kornienko et al. [47] identified VNTR regions containing highly conserved sequences that terminate with a CAT triplet in all sturgeon species studied. TAS elements are located in repeating units that constitute the VNTR. TAS nucleotide sequences are associated with the termination of mtDNA replication. An ineffective DNA repair system and a lack of protective histones in this organelle cause the mutation rate in mitochondrial DNA to be higher than in the DNA nucleus, resulting in VNTRs. Tandem repeats in this region increase the length of the D-loop, and a lower D-loop increases the rate of supercoil formation [43]. This issue has an effect on protein bonding and accessibility as well as on transcription and replication [48, 49] (Fig. 4).

Variation in repeat tandems in this region probably evolved through the process of illegitimate elongation [45]. It occurs during mtDNA replication and is possibly the mechanism mediated by slipped strands and mispairing [50].

We identified conserved CSBD and CSB-1, CSB-2, and CSB-3 sequence blocks with high similarity of CSB sequences from other fishes (Fig. 4). While five conserved block sequences have been reported in the conserved central domain in mammals in the control region, only 3 CSBs are typically found in bony fishes [51–53]. CSB-D is critical in maintaining regulatory performance in CR and is considered the most protected part of CR (95% of its sequence is conserved). The CSB 1, 2, and 3 sequences had the least conserving. Consensus sequences for CSB could be found here:

- CSB-D, TACTGGCATCTGATTAAT;
- CSB-1, TAATAGATAGTGAATGATATAATGACATA;
- CSB-2, CAAACCCCCTACCCCC;
- CSB-3, TGTCAAACCCCAAAAGCA.

In addition, three conserved sequences were found downstream of the protected central conserved domain, and CSB-1 is an AT-rich region following a GACATA conserved motif.

The downstream CSBs are separated by a poly C and are identified by TTA or TA. CSB 2 consists of a sequence with a polyC stretch. These CSBs are involved in forming primer RNA for mtDNA amplification and play a vital role in the RNA switch in DNA synthesis, which begins in the OL region [29, 54].

**Fig. 5:**
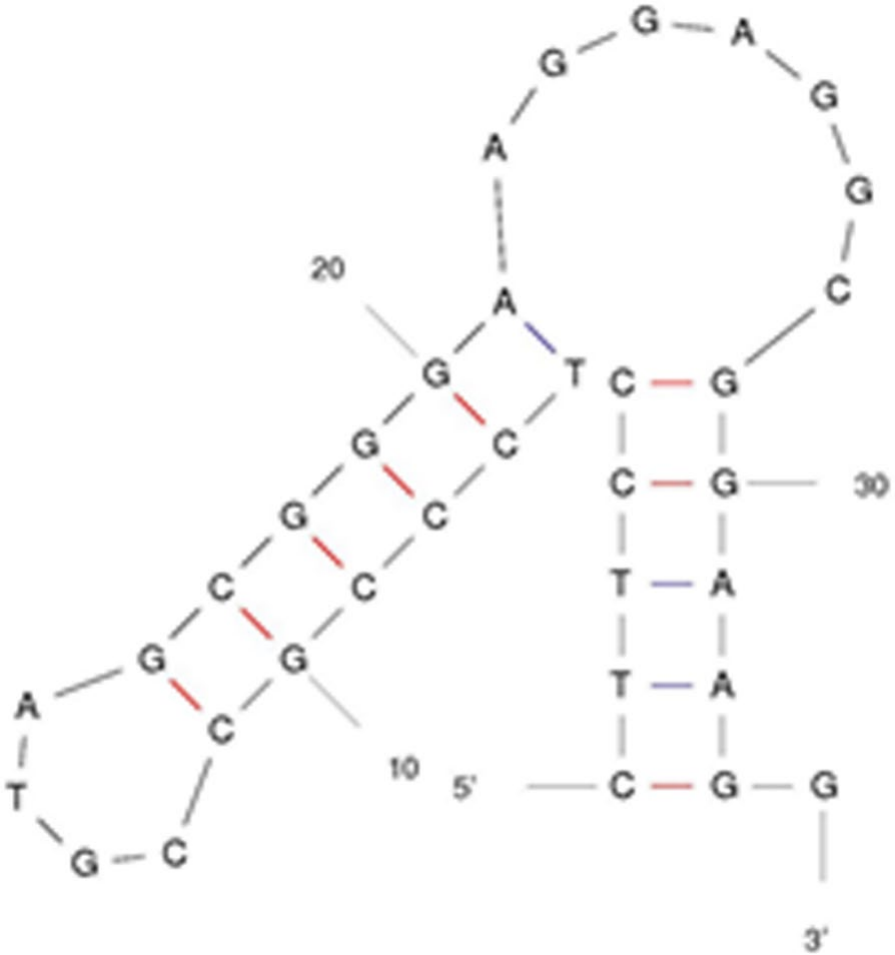
The OL region was found in *Huso huso*

The noncoding OL region is located in a cluster of five tRNA genes (trp (W), Ala (A), Asn (N), Cys (C), and Tyr (Y)). It is called the WANCY region. The OL region has 34 nucleotides and is identified by a stable loop stem structure with a GC-rich stem and a T-rich loop. Two common features of vertebrate OL are the 5' flanking region rich in pyrimidine (stem) and a motif (5ʹ-CTTCCT-3ʹ) found in the stem (Fig. 5).

Both may be related to the accuracy and efficiency of DNA replication in OL, as seen in the human mitochondrial genome [55].

### Phylogenetic analysis

The control regions and the regions that caused the instability in the phylogenetic tree were discarded (the high homogeneity of the genes encoding the protein, due to their high capability in phylogenetic function, were used in the experiment). We performed partition maximum likelihood (ML) using the concatenated nucleotide sequences of the 12 protein-coding genes.

We excluded VNTRs because they are rapidly evolving sequences, which may lead to multiple substitutions at some sites. Therefore high heteroplasy would reduce the resolution of our phylogeny. The sturgeon species created two clades in the phylogenetic trees (Atlantic, Pacific).

**Fig. 6:**
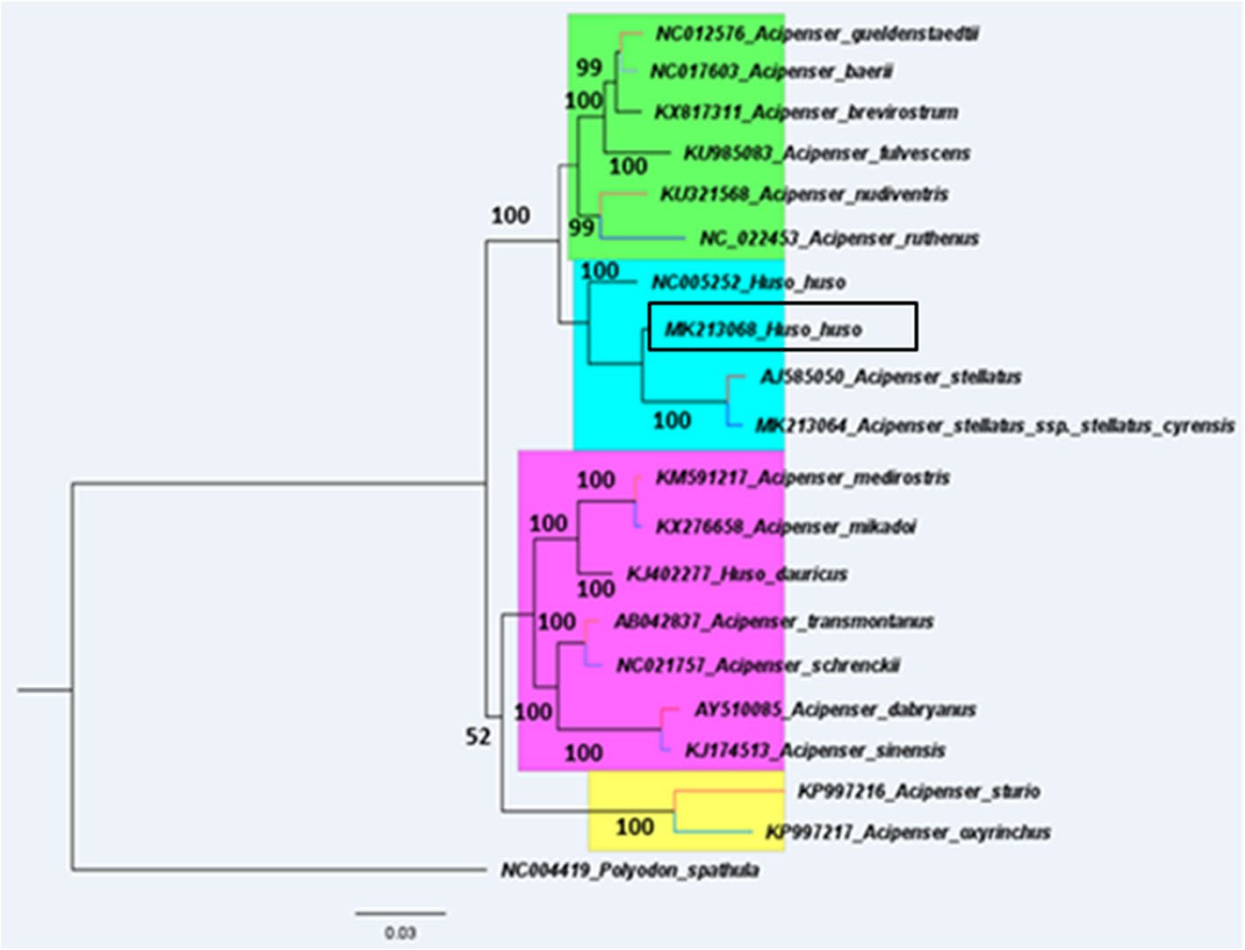
Evolutionary relationship between *Huso huso* and other sturgeon species by a phylogenetic tree

Mitogenomic phylogeny has put *Acipenser stellatus* as a sister group of *Huso huso* with high support (bootstrap 100% and posterior probability 1.00). *Huso huso* similarly formed with high support (bootstrap 100%) a sister group to the remaining species of clade Atlantic. This topology is consistent with the phylogenies of the complete mitochondrial genome of Liao et al. [2], Li et al. [56], and Popovic et al. [57]. This topology is inconsistent with the findings of Mugue et al. [20]. Paraphilic species with *Huso huso*, including *Acipenser guelenstaedtii*, *Acipenser baerii*, *Acipenser nudiventris*, *Acipenser fulvescens*, *Acipenser ruthenus*, *Acipenser brevirotrum*. Our results agree with the taxonomy for sturgeons within the genus and reflected monophilic and paraphilic taxa (Fig. 6) [2, 30, and 61]. The analysis of Nedoluzhko et al. [58] and Sheraliev et al. [40] showed that in the Atlantic clad, *H. huso* was put as a sister group with all remaining *Acipenser* species. Also, Sheraliev et al. [40] confirmed that A. stellatus is closely related to *P. kaufmanni* (Fig. 6). Sheraliev et al. [40] showed that *H. huso* is not closely related to *A. stellatus*, and is an ancestor clade Atlantic species, these results contradict our findings and are consistent with some results [59]. « A. oxyrinchus” and *A. sturio* formed a separate branch in the Pacific clade, clustered as paraphily with the rest species in this clade. *Acipenser guelenstaedtii* and *Acipenser baerii* formed a group monophyletic, whereas *Acipenser nudiventris* and *Acipenser ruthenus* were together monophyletic in the clade Atlantic. *A. transmontanus* and *A. schrenckii* formed group monophyletic while *A. sinensis* and *A. dabryanus* were monophyletic in the clade Pacific. Polyodontidae was used as the outgroup because it was an ancient sturgeon. It is generally believed that the sturgeons originated from the ancient population in Europe, and the early diversity occurred in Asia [40]. Short mitochondrial gene fragments have revealed limitations in complex phylogenetic relationships in many lineages. Many informative sites from longer DNA sequences, such as the complete mitochondrial genome, allow deeper branches and higher levels of relationships to resolve complex relationships. Based on more genetic data, the mitochondrial genome will completely elucidate the phylogenetic relationships and determine a higher lineage level.

### The substitution rates genes

More details showed that the divergence between substitutions in the mitochondrial genome of rRNA and tRNA was less than that in the protein coding genes, indicating the stability of the stem and loop in the rRNA and tRNA. The CR had the most divergence. The divergence ratio varies between the genes encoding the protein, and ATP8, ND3, ND6, Cytb, and ND4 L showed the highest conservation, but ND2 and COII showed the highest divergence. ND2 is a small protein-encoding gene that is amplified by a primer and can be used as a protein-encoding gene with good divergence in barcoding species is much more suitable than COI (the complete gene can be used instead of the partial gene). Applying these new genomic sequences to taxonomic tests of this species will be very useful. It will have achievements for phylogenetic analysis and the study of lineage rearrangements, conservation, and evaluation of biological studies.

We calculated the ratio of nonsynonymous substitutions (Ka) to the rate of synonymous substitution (Ks) (Fig. S2). Our results show that all 13 genes have a Ka/Ks ratio lower than one, indicating a strong selection signal for harmful mutations in all mitochondrial protein genes. However, the mean Ka/Ks ratio (0.1 to 0.001) showed a significant difference between individual genes. The highest rate (ND2, COII) indicates that the purification selection is under minimal pressure.

## Conclusions

We identified the complete mitochondrial genome consisting of 22 tRNAs, two rRNAs, 13 protein-encoding genes, and two OL and D-loop control regions. The VNTR is found in three sites, and a VNTR is between ND6 and Cytb as pseudo-tRNA-Glu. This organization of the genome was separate from other previously reported sturgeon species. These results included increased tRNA in H. huso and a VNTR in the 12 SrRNAs.

The 13 genes encoding proteins show that they are less conserved than rRNA and tRNA. Divergence varies between genes. COII was the highest, while the ATP8 synthetase subunit was the lowest. Observing the divergence rates of these genes allows us to compare them for barcoding. Although COI is commonly used for DNA barcoding, this gene has observed low divergence, indicating that it cannot clearly distinguish very close species. The data analysis showed that ND2 is a better candidate for barcode identification in the grouping. It has a higher percentage of variable sites than COI. Therefore, it can differentiate between newly derived species.

In addition, the smaller size of ND2 makes it easier to use compared to COI. The sequence was amplified entirely using only one pair of primers. Thus, a complete gene instead of a partial gene can be used for barcoding.

Nevertheless, we observed a kind of diversity in this particular species, which included the presence of tandem repeats in the control region and 12S rRNA and tRNA-Glu, resulting from mispairing. There have been reports of pseudo tRNA in several species of fish, but there have been no reports about the presence of repeats in fish in 12S rRNA.

## Methods

### Sampling, PCR amplification, and sequencing

Samples of sturgeon were taken from the waters of Iran on the south coast of the Caspian Sea. Systematic studies were performed to select a random animal specimen to avoid exaggerating the effects and to conform to the conventional statistically significant criteria. Samples were received from three provinces along the south coast of the Caspian Sea (Mazandaran, Golestan, Gilan) in five fishing areas, and samples were collected from the caudal fin. Complete genomic DNA was obtained using the ammonium acetate method [60], and 16 pairs of universal primers overlapping mitochondrial genome fragments were amplified and then sequenced.

Primers and PCRs were performed based on the methods described by Shao et al. [61] (Table S1). One hundred μl of PCR products, along with 50 μl of each forward and reverse primer (10 pmol), were used to determine the sequences of DNA fragments using the Sanger method by the European company of Microsynth.

#### Assembling genome sequences and annotating

The sequencing results were manually corrected and edited using (ChromaSprov.1.42), and then the mitochondrial genome was searched for protein and rRNA using the BLAST tool. http://www.ncbi.nlm.nil.gov/BLAST.cgi, and annotations were performed based on the mitochondrial genome alignment of closely related species in the GenBank database. The obtained sequences from 16 pieces were aligned with Clustal X and then edited with BioEdit software.

The overlapping sequences were achieved, and the results of the contigs were assembled using the SeqMan module of Lasergene 11.0 software (DNASTAR, Madison, WI, USA) and then mapped to reach the complete mitochondrial genome of Huso huso. Sequence results and combined annotations were used to map the species' genome. Most tRNA genes and their secondary structures were predictable by tRNA-scan [62]. The cloverleaf structure was detected by computer.

### Sequence analysis

Nucleotide compositions were obtained using the DNASTAR program, and ATskew and GCskew were calculated using the formula [56].

$$\mathrm{ATskew}=\left[\mathrm A-\mathrm T\right]/\left[\mathrm A+\mathrm T\right]\;\mathrm{and}\;\mathrm{GCstew}\;=\left[\mathrm G-\mathrm C\right]/\left[\mathrm G+\mathrm C\right]$$

Tandem Repeat Finder was used to identify duplicate sequences. The complete mitochondrial genome sequencing of Ka/Ks, the ratio of protein-coding genes, was calculated by PAL2NAL online (http://www.bork.embl.de/pal2nal). The codon usage of 13 protein-coding genes was calculated using Mega 10 software.

### Phylogenetic analysis

In this study, mitogenomes of additional species were obtained from GenBank and aligned using Clustal X. After removing their stop codons to draw the phylogenetic tree, the genes became concatenated. We used European, Chinese, Russian, and American sturgeon species for phylogeny. Sequences of 12 genes encoding proteins were aligned with Clustal X, and minor manual settings were used.

To compare the differences, probability tests were performed. Different models were used for comparison and Determination, and the best model selection based on BIC was determined for 12 protein-coding genes. Finally, GTR + I + G was selected as the best model, and 12 genes based on maximum likelihood and gamma distribution were evaluated using all sites and bootstrap 1000.

### Supplementary Information

**Additional file 1: Fig. S1.** The secondary structure of the variable number of tandem repeats (VNTR) in the 12S rRNA gene. **Fig. S2.** The average Ka/Ks ratio of 13 protein-coding genes Ka/Ks is the nonsynonymous substitution rate (Ka) ratio to the synonymous substitution rate (Ks). **Table S1.** Primer sequences and product size. **Table S2.** The average base composition of 13 protein-coding genes in Huso huso.

## Data Availability

DNA sequence data generated and analyzed in this manuscript were deposited in a public database, NCBI (Accession number: MK213068). https://www.ncbi.nlm.nih.gov/nuccore/MK213068.
