# Supplementary material for: Characterization of the mitochondrial Huso huso genome and new aspects of its organization in the presence of tandem repeats in 12S rRNA
Source: BMC Ecol Evol. 2023 Sep 26;23:55. doi: 10.1186/s12862-023-02166-2 (PMC10521412; doi:10.1186/s12862-023-02166-2)
Supplement: Supplementary file 1 — Additional file 1: Fig. S1. The secondary structure of the variable number of tandem repeats (VNTR) in the 12S rRNA gene. Fig. S2. The average Ka/Ks ratio of 13 protein-coding genes Ka/Ks is the nonsynonymous substitution rate (Ka) ratio to the synonymous substitution rate (Ks). Table S1. Primer sequences and product size. Table S2. The average base composition of 13 protein-coding genes in Huso huso. [file 12862_2023_2166_MOESM1_ESM.docx]

| Product  size  (bp) | Primer sequences (5'–3') | Annealing  temperatue  (°C) | Primer name | No. of  primer  pair |
| --- | --- | --- | --- | --- |
| 1289 | F-5ꞌ- ACAAACCCCCTACCCCCTTA-3ꞌ  R-5ꞌ- CCTTTTCTATCGCCTATACTGG- 3ꞌ | 58 | Asc-F16605  Asc-R1216 | 1 |
| 1202 | F-5ꞌ-TTGGAACAACCAAAATGTAGCTC-3ꞌ  R-5ꞌ- ACGAGCAGGTCAATTTCACTGA-3ꞌ | 58 | Asc-F1020  Asc-R2222 | 2 |
| 1263 | F-5ꞌ- GAAGGTAGCGTAATCACTTGTC-3 ꞌ  R-5ꞌ-AGGGTGAAATTGCCAGTGAAGA-3ꞌ | 58 | Asc-F2120  Asc-R3383 | 3 |
| 1123 | F-5ꞌ- TACTCAATCTTAGGCTCCGG-3ꞌ  R-5ꞌ- CTAAGGCTGTGGGGTTGGA-3ꞌ | 58 | Asc-F3238  Asc-R4361 | 4 |
| 1228 | F-5ꞌ- CCTAGGAATTGGAACTACCCT-3ꞌ  R-5ꞌ- GTAGATGAATGCTCGCTGGATA-3ꞌ | 58 | Asc-F4111  Asc-R5339 | 5 |
| 1352 | F-5ꞌ- ACATCTTCTGAATGCAACCCAG-3ꞌ  R-5ꞌ-TTAAGCCTCCCACTGTGAATAG-3ꞌ | 58 | Asc-F5219  Asc-R6571 | 6 |
| 1208 | F-5ꞌ- ATGGACGTAGACACACGGG-3ꞌ  R-5ꞌ- GGGATTATGTAGGAGTCGAAG-3ꞌ | 58 | Asc-F6400  Asc-R7608 | 7 |
| 1254 | F-5ꞌ- GGACACCAATGATACTGAAGT-3ꞌ  R-5ꞌ-GAATTATTGCTACGGCTACTTC-3ꞌ | 58 | Asc-F7538  Asc-R8792 | 8 |
| 1147 | F-5ꞌ-ACTCACGGCAAACCTAACTG-3ꞌ  R-5ꞌ-ACTGCTAGGATGCAGGATAG-3ꞌ | 58 | Asc-F8614  Asc-R9761 | 9 |
| 1232 | F-5ꞌ-GATGAGGATCATAACCTTTCTAG-3ꞌ  R-5ꞌ-TGCGTTAAGTCGTTCAGTTTGG-3ꞌ | 58 | Asc-F9617  Asc-R10849 | 10 |
| 1204 | F-5ꞌ-CTAGCAAGCCAAAACCACATC-3ꞌ  R-5ꞌ-GGGTTTAGCTCCCATCTTTAG-3 | 58 | Asc-F10646  Asc-R11850 | 11 |
| 1286 | F-5ꞌ-ACACACGAGAACACCTACTCA-3ꞌ  R-5ꞌ-TGGGGTTGGTTTAAGCCGAT-3ꞌ | 58 | Asc-F11703  Asc-R12989 | 12 |
| 1135 | F-5ꞌ-CAGCAATAGAAGGTCCAACAC-3ꞌ  R-5ꞌ-GGCTCTTCGTGCTGTTTAG-3ꞌ | 58 | Asc-F12725  Asc-R13860 | 13 |
| 1247 | F-5ꞌ-CAACTCACCTAATTGACCAAAC-3ꞌ  R-5ꞌ-ACTAGTGTGTCGCCGATGTA-3ꞌ | 58 | Asc-F13670  Asc-R14917 | 14 |
| 1095 | F-5ꞌ-CTGACATTTCAACAGCCTTCTC-3ꞌ  R-5ꞌ-TCTTAGCTTTGGGAGTTAAGGG-3 | 58 | Asc-F14605  Asc-R15700 | 15 |
| 1105 | F-5ꞌ-AACTGCCCTAGTAGCTTAGAC-3ꞌ  R-5ꞌ-GACAAGTCAGTCCTGCTTTTG-3ꞌ | 58 | Asc-F15573  Asc-R16678 | 16 |

**Table S1. 1.**Primers sequences and product size

| **Genes** | Total | | | | First position | | | | Second position | | | | Third position | | | |
| --- | --- | --- | --- | --- | --- | --- | --- | --- | --- | --- | --- | --- | --- | --- | --- | --- |
|  | **A%** | **T%** | **G%** | **C%** | **A%** | **T%** | **G%** | **C%** | **A%** | **T%** | **G%** | **C%** | **A%** | **T%** | **G%** | **C%** |
| **ND1** | 29.1 | 25.4 | 13.8 | 31.6 | 24.9 | 21.5 | 25.2 | 28.3 | 18.8 | 40.9 | 11.4 | 28.9 | 43.7 | 13.8 | 4.9 | 37.6 |
| **ND2** | 31.7 | 21.9 | 13.4 | 33.0 | 36.4 | 15.5 | 20.6 | 27.5 | 16.7 | 37.4 | 11.5 | 34.5 | 40.0 | 12.9 | 8.0 | 37.1 |
| **COI** | 24.3 | 27.5 | 19.6 | 28.4 | 24.3 | 22.4 | 31.4 | 22.0 | 18.4 | 40.0 | 15.3 | 26.4 | 30.4 | 20.3 | 12.2 | 36.7 |
| **COII** | 30.8 | 25.8 | 17.1 | 26.3 | 23.4 | 19.0 | 31.2 | 26.4 | 28.3 | 38.3 | 10.9 | 22.6 | 40.9 | 20.0 | 9.1 | 30.0 |
| **ATP8** | 32.7 | 25.0 | 11.9 | 30.4 | 33.9 | 23.2 | 12.5 | 30.4 | 25.0 | 33.9 | 12.5 | 28.6 | 39.3 | 17.9 | 10.7 | 32.1 |
| **ATP6** | 26.8 | 25.3 | 13.4 | 34.4 | 28.5 | 13.2 | 21.9 | 36.4 | 14.5 | 47.8 | 11.8 | 25.9 | 37.3 | 14.9 | 7.0 | 40.8 |
| **COIII** | 26.4 | 25.4 | 17.5 | 30.8 | 20.2 | 23.7 | 29.4 | 26.7 | 21.0 | 36.3 | 16.8 | 26.0 | 37.9 | 16.1 | 6.1 | 39.8 |
| **ND3** | 22.9 | 27.8 | 17.8 | 31.5 | 16.2 | 25.6 | 29.9 | 28.2 | 15.5 | 43.1 | 12.9 | 28.4 | 37.1 | 14.7 | 10.3 | 37.9 |
| **ND4L** | 24.6 | 24.9 | 14.5 | 36.0 | 21.2 | 23.2 | 23.2 | 32.3 | 16.2 | 39.4 | 14.1 | 30.3 | 36.4 | 12.1 | 6.1 | 45.5 |
| **ND4** | 28.2 | 24.4 | 15.6 | 31.6 | 29.7 | 18.9 | 21.9 | 29.5 | 16.3 | 40.7 | 14.8 | 28.2 | 38.5 | 13.7 | 10.2 | 37.2 |
| **ND5** | 30.7 | 24.2 | 13.0 | 32.1 | 34.7 | 18.9 | 20.5 | 25.9 | 20.4 | 39.4 | 12.1 | 28.2 | 37.0 | 14.3 | 6.4 | 42.3 |
| **Cytb** | 27.0 | 26.3 | 15.3 | 31.4 | 24.9 | 23.4 | 24.9 | 26.8 | 20.8 | 40.5 | 13.7 | 25.0 | 35.3 | 15.0 | 7.4 | 42.4 |
| **ND6** | 11.9 | 41.0 | 35.1 | 12.1 | 9.2 | 34.5 | 45.9 | 10.9 | 13.2 | 20.7 | 46.0 | 20.1 | 13.2 | 42.5 | 39.1 | 5.2 |

**Table S2.** The average base composition of 13 protein -coding genes in *Huso huso*

| **AA** | **codon** | **RSCU** | **AA** | **codon** | **RSCU** |
| --- | --- | --- | --- | --- | --- |
| **Phe(F)** | TTT | 0.97 | **His(H)** | CAT | 0.87 |
|  | TTC | 1.03 |  | CAC | 1.13 |
| **Asn(N)** | AAT | 093 | **Cys(C)** | TGT | 1.02 |
|  | AAC | 1.07 |  | TGC | 0.98 |
| **Lys(K)** | AAA | 1.62 | **Arg(R)** | CGT | 0.35 |
|  | AAG | 0.38 |  | CGC | 1.05 |
| **Leu(L)** | TTA | 1.05 |  | CGA | 1.25 |
|  | TTG | 0.56 |  | CGG | 1.1 |
|  | CTT | 1.16 | **Ser(S)** | TCT | 0.79 |
|  | CTC | 1.12 |  | TCC | 1.78 |
|  | CTA | 1.35 |  | TCA | 1.35 |
|  | CTG | 0.76 |  | TCG | 0.54 |
| **Pro(P)** | CCT | 0.95 |  | AGT | 0.62 |
|  | CCC | 1.35 |  | AGC | 0.97 |
|  | CCA | 1.31 | **Asp(D)** | GAT | 0.81 |
|  | CCG | 0.39 |  | GAC | 1.19 |
| **Thr(T)** | ACT | 1.03 | **Glu(E)** | GAA | 1.37 |
|  | ACC | 1.43 |  | GAG | 0.27 |
|  | ACA | 1.23 | **Gly(G)** | GGT | 0.66 |
|  | ACG | 0.31 |  | GGC | 1.23 |
| **Val(V)** | GTT | 0.13 |  | GGA | 1.16 |
|  | GTC | 0.83 |  | GGG | 1.06 |
|  | GTA | 1.38 | **Ile(I)** | ATT | 0.87 |
|  | GTG | 0.96 |  | ATA | 1.13 |
| **Ala(A)** | GCT | 0.81 | **Trp(W)** | TGG | 0.7 |
|  | GCC | 1.8 |  | TGA | 1.3 |
|  | GCA | 0.94 | **Gln(Q)** | CAA | 1.35 |
|  | GCG | 0.35 |  | CAG | 0.65 |
| **Met(M)** | ATG | 1.26 | **Tyr(Y)** | TAT | 0.88 |
|  | TAT | 0.74 |  | TAC | 1.12 |

**Table S3.** Codon usage in the PCGs of *Huso huso*


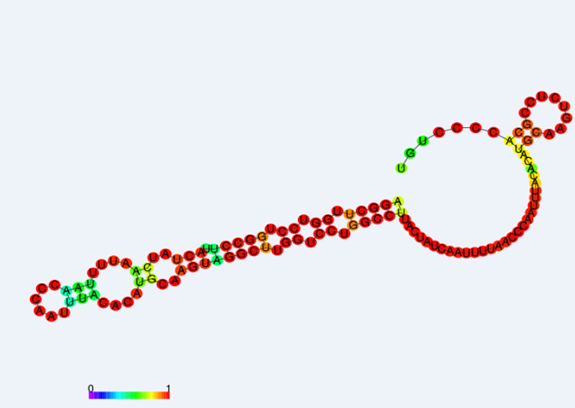

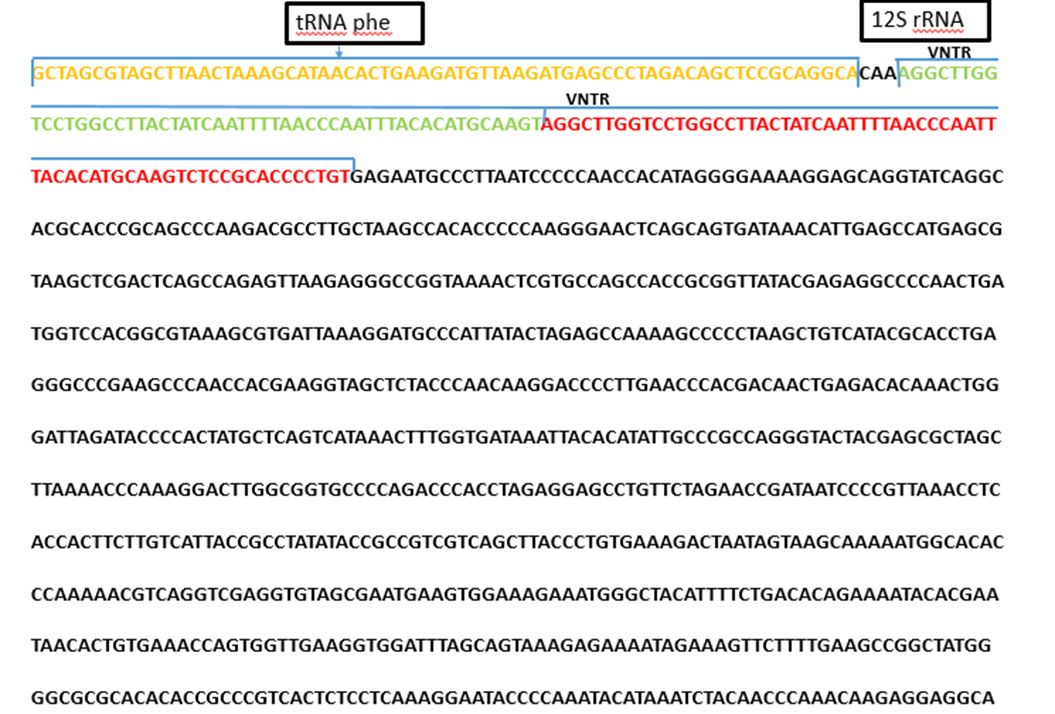


**Fig S1.** The secondary structure of the Variable number of tandem repeats (VNTR) in 12S rRNA gene

**
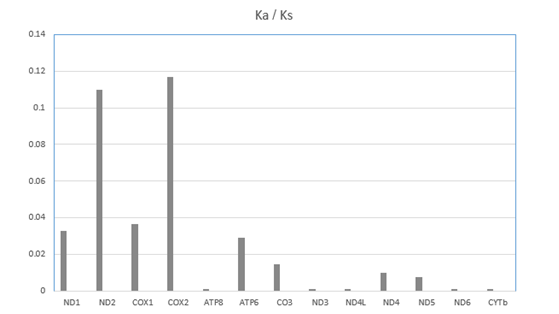
**

**Fig S2.** The average Ka/Ks ratio of 13 protein-coding genes Ka/Ks is the ratio of the nonsynonymous substitution rate (Ka) to the synonymous substitution substitution rate (Ks).
